# Self-reported food allergy in the elderly – Lifetime prevalence and clinical features in older adults seeking allergy care in Brazil

**DOI:** 10.1016/j.waojou.2026.101472

**Published:** 2026-09-28

**Authors:** José Laerte Boechat, Bernardo Sousa-Pinto, Fábio Kuschnir, Emanuel Sarinho, José Rodrigo de Moraes, Luis Taborda-Barata, Carlos Lozoya-Ibáñez, José Luis Delgado, Fátima Rodrigues Fernandes, Daniella Moore, Décio Medeiros, Maria Elisa Bertocco Andrade, Myrthes Toledo Barros, Mário Gustavo de Aranda Pacheco, Maria Letícia Chavarria, Valéria Soraya de Farias Sales, Liza Souza Brito, Priscilla Filippo Alvim de Minas Santos, Isabela Maria Anselmo Ribeiro Simões, Albertina Capelo, José Dilbery Oliveira da Silva, Valéria Botan Gonçalves, Ana Luiza Ribeiro Bard de Carvalho, Camila Martins Chieza, Bianca da Mota Pinheiro, Daniele Lemos, Mara Morelo Félix, Lika Nishimori, José Luiz Rios, Maria do Socorro Viana Silva de Sá, Nonayra Oliveira, Gabriela Dias, Zulmira Ernestina Pereira Lopes, Vanessa Bosi Bissi Denadai, Laura Gottschall da Silva, Elaine Alves da Rocha, Tatiana Andrade, Fabrício Prado Monteiro, Fernanda Lugão Campinhos, Magna Patrícia Saiter Coutinho, Manuela Rodrigues Coimbra, Lidiane Simões, Mariana Castiglioni, Eliane Miranda da Silva, Ludmila Franco, Raquel Prudente de Carvalho Baldaçara, Marcelo Jeferson Zella, Norma Rubini, Ekaterini Goudouris, Gustavo Wandalsen, Dirceu Solé

**Affiliations:** a Allergy and Immunology Service, Department of Internal Medicine, Faculty of Medicine, Fluminense Federal University, Niterói, Brazil; b Basic and Clinical Immunology Service, Department of Pathology, Faculty of Medicine, University of Porto, Porto, Portugal; c RISE-Health, Faculty of Medicine, University of Porto, Porto, Portugal; d Brazilian Association of Allergy and Immunology (ASBAI), São Paulo, Brazil; e MEDCIDS – Department of Community Medicine, Information and Health Decision Sciences, Faculty of Medicine, University of Porto, Porto, Portugal; f Allergy and Immunology Service, Department of Pediatrics, Faculty of Medical Sciences, University of the State of Rio de Janeiro, Rio de Janeiro, Brazil; g Allergy and Immunology Service, Faculty of Medicine, Federal University of Pernambuco, Recife, Brazil; h Institute of Mathematics and Statistics, Department of Statistics, Fluminense Federal University, Niterói, Brazil; i RISE- Health, Faculty of Health Sciences, University of Beira Interior, Covilhã, Portugal; j UBIAir - Clinical & Experimental Lung Centre, University of Beira Interior, Covilhã, Portugal; k Department of Immunoallergology, Cova da Beira University Hospital Center, Covilhã, Portugal; l Allergy Department, Unidade Local de Saúde of Castelo Branco, Castelo Branco, Portugal; m Allergy Unit, CUF Porto Hospital and Institute, Porto, Portugal; n Allergy and Immunology Service, Hospital Do Servidor Publico Estadual, Institute of Medical Assistance to State Public Servants, São Paulo, Brazil; o Discipline of Allergy and Clinical Immunology, Hospital Das Clinicas, Faculty of Medicine, University of São Paulo, São Paulo, Brazil; p Institute of Tropical Pathology and Public Health, Goiás Federal University, Goiânia, Brazil; q Discipline of Clinical Immunology, Rio Grande Do Norte Federal University, Natal, Brazil; r Allergy and Immunology Unit, Hospital Municipal Jesus, Rio de Janeiro, Brazil; s Allergy Unit, Hospital Estadual de Américo Brasiliense, Américo Brasiliense, Brazil; t Allergy and Immunology Service, Hospital Universitário Gaffrée e Guinle, Federal University of the State of Rio de Janeiro, Rio de Janeiro, Brazil; u Department of Pediatrics, Faculty of Medicine, Federal University of Campina Grande, Cajazeiras, Brazil; v Hospital da Criança de Brasília José Alencar, Distrito Federal, Brazil; w Medical Specialities Center Dr Amur Ferreira, Araucária, Brazil; x Allergy Outpatient Unit, Policlinica Metropolitana Do Estado Do Pará, Belém, Brazil; y Allergy Unit, Policlinica Universitaria Piquet Carneiro, University of the State of Rio de Janeiro, Rio de Janeiro, Brazil; z Allergy and Immunology Service, Hospital Federal Dos Servidores Do Estado, Rio de Janeiro, Brazil; aa Allergy and Immunology Service, Hospital Central Do Exército, Rio de Janeiro, Brazil; ab Allergy Unit, Petrópolis Faculty of Medicine, Petrópolis, Brazil; ac Discipline of Clinical Immunology, Faculty of Medicine, Federal University of Campina Grande, Campina Grande, Brazil; ad Basic Immunology and Genetics, Faculty of Medicine, Vila Velha University, Vila Velha, Brazil; ae Reference Center for Asthma, Allergy and Immunology, Santa Casa de Misericórdia de Vitória, Vitória, Brazil; af Allergy Unit, Hospital Ministro Costa Cavalcanti, Foz Do Iguaçu, Brazil; ag Allergy Outpatient Unit, Hospital Infantil Albert Sabin, Fortaleza, Brazil; ah Allergy and Immunology Unit, Faculty of Medicine, Federal University of Tocantins, Palmas, Brazil; ai Department of Pediatrics, Faculty of Medicine, Rio de Janeiro Federal University, Rio de Janeiro, Brazil; aj Discipline of Allergy, Clinical Immunology and Rheumatology, Escola Paulista de Medicina, Federal University of São Paulo, São Paulo, Brazil

**Keywords:** Elderly, Food allergy, Prevalence, Self-reported questionnaire

## Abstract

**Background:**

Food allergy (FA) is a growing concern worldwide. However, most epidemiological studies focus on childhood or young adults, so that FA in the elderly remains poorly characterised. This study aimed to investigate the lifetime prevalence of self-reported FA among older Brazilians seeking allergy care, and possible associations with sociodemographic and clinical characteristics.

**Methods:**

Cross-sectional study using a self-reported questionnaire (developed, standardised and validated in Portuguese) applied to a non-probabilistic sample of elderly people (≥60 years) living in Brazil and recruited by their attending physicians in public or private care units. Multivariable regression models were applied to estimate the association of sociodemographic or clinical characteristics with self-reported FA.

**Results:**

We included 632 individuals (69.8% female; average age: 70.2 years) from all 5 Regions of Brazil. The prevalence of self-reported FA was 18.8% (95%CI = 16.0%–22.1%), based on the affirmative answer to the question: “have you ever had an allergic reaction to any food?”. A total of 9.0% (7.0%–11.5%) reported having had a medical diagnosis of FA, while 5.5% (4,0%-7,6%) reported having had an immediate reaction (<2 h) and 2 or more episodes with the same food. The foods most frequently associated were shellfish, milk and fresh fruits. The most frequent clinical manifestations were urticaria and gastrointestinal symptoms. In multivariable analysis, we found an association between FA and female sex, as well as personal and family history of allergy.

**Conclusions:**

The lifetime prevalence of self-reported FA among elderly individuals recruited in allergy/immunology clinical settings in Brazil was high, being more common in women and in those with personal or family history of allergy.

## Introduction

Epidemiological studies indicate that the prevalence of food allergy (FA) is increasing globally, exhibiting significant variability between and within countries, across different age groups, and depending on the research method used for diagnosis. However, for a long time, FA was regarded almost exclusively as a pediatric issue. In recent decades, possibly due to environmental factors and lifestyle changes, there has been a significant increase in the prevalence of FA in adults and in the elderly, with peculiar pathogenic mechanisms and symptoms that often make diagnosis even more challenging in the latter group.^1^, ^2^, ^3^, ^4^ The mechanisms include many factors, beyond immunosenescence itself, that influence the loss of immune tolerance and greater susceptibility to FA in the elderly: changes in gut microbiota composition, decline in intestinal barrier function, and micronutrient deficiencies. Intrinsically, in the senescent immune system, notable features include the presence of dysfunctional dendritic cells, reduced synthesis of secretory IgA, a shift in the adaptive immune response to a Th2 pattern, and decreased function of regulatory T cells.^5^^,^^6^

Furthermore, the presence of comorbidities, concurrent use of multiple medications (polypharmacy), medication side effects, chronic alcohol consumption, chronic infections, and the decline of physiological functions should be considered age-associated risk factors for sensitisation to food allergens and taken into account in the diagnosis and management of elderly individuals with FA. These factors can also alter the clinical manifestations of FA, which may go unnoticed, thereby contributing to the underdiagnosis of the condition in this age group.^7^^,^^8^

Despite FA in the elderly being increasingly recognised as a relevant concern, significant knowledge gaps remain. One of these gaps concerns the prevalence of FA in the elderly. In fact, some meta-analyses have estimated the prevalence of FA in adults to vary between 3.5% and 35% when only based on self-report, and between 2% and 4% when studies include more stringent additional criteria such as positive skin prick test (SPT) and/or food-specific IgE levels or the gold standard of double-blind placebo-controlled food challenge (DBPCFC).^9^, ^10^, ^11^ However, few studies have been conducted on the prevalence of FA in elderly individuals. Such few studies (all in North America and Europe) have found prevalence rates ranging from 4.4% to 19.1%.^12^, ^13^, ^14^, ^15^ The wide disparity in these prevalence values observed in epidemiological studies of FA across distinct age groups may result from the use of different research methodologies: in this context, the operational definition of FA adopted in the study is pivotal to ensuring comparability between studies.^9^, ^10^, ^11^^,^^14^^,^^16^^,^^17^ Additionally, regional differences may partly account for the observed variations in the prevalence of FA. There are currently no epidemiological studies on the prevalence of FA (or the foods most commonly implicated) in this age group in Brazil or in other Latin American countries.

In the present study, we aimed to estimate the lifetime prevalence of self-reported FA among older Brazilians recruited in clinical settings (allergy/immunology services), as well as to identify the most frequently implicated foods and symptoms, and to assess associations with sociodemographic and clinical characteristics.

## Methods

We performed a cross-sectional study using a questionnaire developed, standardised and validated in Portuguese^18^ to estimate the prevalence of self-reported FA and identify implicated foods. This questionnaire was applied to a non-probabilistic sample of elderly people (≥60 years) living in Brazil and recruited sequentially and independently of their current health complaint by their attending physician in public or private specialised care units. The patients were informed that the questionnaire was part of a research protocol on food allergies in the elderly. The manuscript was written according to the recommendations of the STROBE checklist for cross-sectional studies.^19^

### Cross-cultural adaptation of the questionnaire

Since the original questionnaire^18^ was developed and validated in European Portuguese, the translation phase was omitted, and only the verification of its adequacy to the Brazilian cultural context and lifestyle was performed. Thus, a group of experts from the Brazilian Association of Allergy and Immunology (ASBAI) identified 25 specialists in allergy and immunology from across Brazil, with expertise in food allergy, and invited them to participate in this phase of the questionnaire's cultural adaptation. They were asked about the clarity of the questions and their ability to distinguish individuals with possible FA-related conditions. Foods identified as uncommon in Brazil and the terms less frequently used in Brazilian Portuguese were replaced with more appropriate ones, resulting in the final instrument applied in the present study^20^ (Suppl Figure 1).

### Sampling

We employed a convenience sampling method, selecting a sample size that matched the size obtained if a simple stratified sampling plan, without replacement, by Brazilian states had been used. With a 95% confidence level, a maximum absolute sampling error of 2%, and the assumption that the prevalence of FA is no higher than 10%, we calculated that 865 elderly individuals would be needed for the sample.

The total sample was distributed using the proportional allocation method across the Brazilian states, according to the latest Demographic Census by the Brazilian Institute of Geography and Statistics (IBGE), which estimated at 32,113,490 the distribution of elderly individuals aged 60 or older in the general population (Table 1).

**Table 1:** Distribution of the elderly population in Brazil by Major Regions.

Region population	Age group (years)	Men	Women	Total per age group	Total per region	Percentage of elderly population in Brazil (%)
Southeast84,840113	60–69 70–79 ≥80	3,770,978 1,940,355 784,013	4,548,753 2,571,150 1,352,282	8,319,731 4,511,505 2,136,295	14,967,531	46.6
Northeast54,658,515	60–69 70–79 ≥80	1,941,743 1,080,101 494,558	2,291,237 1,354,546 752,695	4,232,980 2,434,647 1,247,253	7,914,880	24.6
South29,937,706	60–69 70–79 ≥80	1,373,150 710,371 261,380	1,589,236 899,446 437,346	2,962,386 1,609,817 698,726	5,270,929	16.4
Central-west16,289,538	60–69 70–79 ≥80	584,602 289,601 117,783	660,927 340,257 157,907	1,245,529 629,858 275,690	2,151,077	6.7
North17,354,884	60–69 70–79 ≥80	523,413 252,708 100,997	536,582 267,380 127,993	1,059,995 520,088 228,990	1,809,073	5.6

Brazilian population in the last census (2022): 203,080,756.

https://cidades.ibge.gov.br/brasil/panorama

The eligibility criteria included patients aged 60 years or older, of both sexes, who attended an outpatient allergy and immunology consultation in public or private services. The elderly were recruited sequentially during the outpatient consultations, without prior selection, and regardless of the reason for the current consultation. Those who agreed to participate signed the informed consent form and were subsequently interviewed by the researcher using the questionnaire, in order to facilitate comprehension, health literacy and avoid incomplete responses.

The study involved 8 participating centers located in public hospitals in the Northeast and Southeast regions of Brazil (where 70% of Brazilian elderly live, Table 1), as well as all active members of the ASBAI, present in all Brazilian states, who were invited to take part in the research. The recruitment period was from June 2022 to August 2023.

### Criteria to define the prevalence of self-reported FA in the study

Based on the responses to the questionnaire and their combinations, we used 3 criteria to define the prevalence of self-reported FA in the present study.

1. General prevalence of self-reported FA: based on the affirmative response to the question “Have you ever had an allergic reaction to any type of food?”
2. Self-reported FA medical diagnosis: based on the affirmative response to both the questions “Have you ever had an allergic reaction to any type of food?” plus “Have you ever been diagnosed with FA by a physician?”
3. Probable FA: for a patient to be classified as having a self-reported probable food allergy, all the following requirements needed to be met:
  a. Self-reported FA medical diagnosis defined by an affirmative response to both questions “Have you ever had an allergic reaction to any type of food?” and “Have you ever been diagnosed with food allergy by a physician?”;
  b. Reporting of 2 or more similar episodes with the same food, defined by the answer “2 or more” to the question “How many similar episodes have you had with the same food?”;
  c. Self-reported immediate reaction defined by the answer “minutes to 2 h” to the question “How long did it take for reactions to occur after ingestion of the food?”.

Additionally, the prevalence of food-induced anaphylaxis in the studied population was evaluated through the positive answer to the question “What type of reaction did you have? (Anaphylactic shock)”.

### Definition of some terms included in the questionnaire

The researchers were instructed to explain the following terms to the patients in a standardised manner during the interview.

1. Gastrointestinal (abdominal) symptoms: vomiting, diarrhea, abdominal pain.
2. Oral allergy syndrome (OAS): immediate reaction, restricted to the oral mucosa, with itching and swelling of the lips, tongue, palate, and/or pharynx. Symptoms usually begin within minutes and gradually disappear within 1 h after contact with the food.
3. Respiratory symptoms: any respiratory symptoms, excluding isolated nasal manifestations, assessed separately.
4. Contact dermatitis: development of eczema lesions associated with contact with the food.
5. Anaphylaxis: the questionnaire included the option “anaphylactic shock”, that is a narrower term, may be variable interpreted by respondents and does not strictly correspond to current clinical criteria for anaphylaxis. In line with this, the researchers were instructed to explain anaphylaxis to the patients as a generalized reaction, involving more than 1 system (skin, respiratory, gastrointestinal, cardiovascular or neurological).
6. Molluscs: mussel, octopus, squid.
7. Latex group: kiwi, banana, mango, papaya, fig, and tomato.
8. Regional foods: typical regional foods not included in the list presented in the questionnaire.

### Statistical analysis

The data are presented as absolute and relative frequencies, and comparisons were done using parametric or non-parametric tests, depending on the nature and distribution of the variables. Clustering by center or physician was not performed. In statistical tests, a significance level of 5% was adopted.

To compute the prevalence of self-reported FA, self-reported FA medical diagnosis and probable FA, correcting for sample imbalances in the sex, age and residence region of the participants, we computed multilevel models with poststratification.^21^^,^^22^ Such models enabled an estimation of the expected prevalences, and considered the populational sex, age and region distribution of elderly people in Brazil (ie, the number of males and females of each age group and in each geographical region of Brazil). In detail, we started by estimating a hierarchical logistic regression model in which the presence of FA was the dependent variable, and there were random effects corresponding to the sex, age group and region of the participants. Subsequently, we computed the predicted probabilities for each combination of sex, age group and region. Finally, we applied the predicted probabilities to the populational number of individuals of each sex, age group and region (post-stratification), so that we were able to obtain adjusted estimates for Brazil. We assessed uncertainty by performing 1000 simulations and computing the mean of the coefficients of the multilevel models with poststratification of these 1000 random samples.

Poisson log-linear regression models with a robust variance estimator were applied to estimate the association between sociodemographic and clinical characteristics and 3 self-reported FA outcomes: general, medically diagnosed and probable. The Wald test was used to evaluate the significance of the association between these characteristics and the study outcomes. We started by conducting univariable regression models. Variables displaying a marginal association (*p* < 0.20) with the outcomes in univariable models were included in a multivariable model. We expressed our results as prevalence ratios (PR) with their respective 95% confidence intervals. Statistical analyses were performed using the software R (version 4.5.1).

The study was approved by the Ethics and Research Committee, Hospital Universitário Antonio Pedro, Universidade Federal Fluminense, Niteroi, Brazil (CAAE: 47781421.8.0000.5243).

## Results

### Characteristics of the study population

Between June 2022 and August 2023, we included 632 individuals (69.8% females) from all 5 Brazilian regions (14 Brazilian states) (Table 2). All elderly individuals interviewed were included in the study, with no losses due to questionnaire completion errors, inconsistencies or a lack of signature on the informed consent form. However, the centers did not systematically record the total number of patients approached and refusals, which precludes calculation of a formal acceptance rate and limits assessment of potential non-response bias. Most of the included participants (77.1%) lived in the Southeast region. The average age of the elderly participants included in the study was 70.2 ± 7.3 years (Table 2).

**Table 2:** Sociodemographic characteristics of the Brazilian elderly interviewed (total and subgroup with self-reported food allergy).

Sociodemographic characteristics	All population (n = 632)		Self-reported food allergy (n = 119)	
	n	% (95% CI)^a^	n	% (95% CI)^a^
***Major brazilian regions***				
North	6	0.9 (0.4–2.1)	3	2.5 (0.9–7.2)
Northeast	86	13.6 (11.2–16.5)	20	16.8 (11.2–24.5)
Central-west	40	6.3 (4.7–8.5)	7	5.9 (2.9–11.6)
Southeast	487	77.1 (73.6–80.2)	86	72.3 (63.6–79.5)
South	13	2.1 (1.2–3.5)	3	2.5 (0.9–7.2)
***Type of health service***				
Private	412	65.2 (61.4–68.8)	69	58.0 (49.0–66.5)
Public	220	34.8 (31.2–38.6)	50	42.0 (33.5–51.0)
***Gender***				
Female	441	69.8 (66.1–73.2)	94	79.0 (70.8–85.3)
Male	191	30.2 (26.8–33.9)	25	21.0 (14.7–29.2)
***Age group***				
60–69 years	332	52.5 (48.6–56.4)	72	60.5 (51.5–68.8)
70–79 years	218	34.5 (30.9–38.3)	33	27.7 (20.5–36.4)
80 years or more	82	13.0 (10.6–15.8)	14	11.8 (7.1–18.8)
***Age (mean ± SD)***		70.2 ± 7.3		69.1 ± 7.1
Female	441	69.7 ± 7.3	94	68.9 ± 7.0
Male	191	71.4 ± 7.3	25	69.3 ± 7.5
***Educational level***				
No education or, at most, an elementary education completed	214	33.9 (30.3–37.6)	37	31.1 (23.5–39.9)
High school or at most incomplete higher education	253	40.0 (36.3–43.9)	48	40.3 (32.0–49.3)
University level and above	165	26.1 (22.8–29.7)	34	28.6 (21.2–37.3)

a Data expressed in % except if specified otherwise; 95% CI = 95% confidence interval (overlapping CIs between the 2 groups of elderly indicate no significant difference at the 5% level); SD = standard deviation

### Prevalence of food allergy

The general lifetime prevalence of self-reported FA was 18.8% (95% CI: 16.0%–22.1%). The prevalence of self-reported FA medical diagnosis was 9.0% (95% CI: 7.0%–11.5%), while that of probable FA was 5.5% (95% CI: 4.0%–7.6%) (Table 3). Considering the results of the multilevel models with post-stratification, we observed a prevalence of self-reported FA of 18.2% (95% CI: 15.2%–21.3%), self-reported FA medical diagnosis of 8.4% (95% CI: 6.4%–10.6%) and that of probable FA of 5.4% (95% CI: 3.8%–7.2%).

**Table 3:** Prevalence, foods involved and clinical manifestations according to the criterion used to define food allergy in the elderly.

	Self-reported prevalence, % (n), 95% CI	Multiple foods involved % (n)	Most frequently involved foods % (n)	Most reported clinical manifestations % (n)	Anaphylaxis (% (n)/food most frequently involved)
General prevalence of self-reported food allergy	18.8 (119), 16.0–22.1	47.9 (57)	Shellfish: 46.2 (55)Fresh fruits^a^: 27.8 (33)^b^ Milk^c^: 19.3 (23)	Urticaria: 38.0 (45) GI symptoms: 15.9 (19) OAS: 9.3 (11)	5.5 (7)/shellfish
Self-reported food allergy medical diagnosis	9.0 (57), 7.0–11.5	57.9 (33)	Shellfish: 64.9 (37)Fresh fruits: 33.4 (19) Milk: 15.8 (9)	Urticaria: 39.4 (22) Respiratory: 11.1 (6) GI symptoms: 10.6 (6)	6.6 (4)/shellfish
Probable food allergy	5.5 (35), 4.0–7.6	68.6 (24)	Shellfish: 68.6 (24)Fresh fruits: 42.8 (15) Milk: 22.9 (8)	Urticaria: 34.5 (12) GI symptoms: 13.7 (5) Respiratory: 11.9 (4)	7.1 (2)/shellfish

GI: gastrointestinal; OAS: oral allergy syndrome.

a Fresh fruits: latex group (kiwi, banana, mango, papaya, figs, tomatoes) + other than those in the latex group.

b Report of isolated OAS symptoms associated with the ingestion of fresh fruits (%/n): 18.2/6 (general); 15.8/3 (medical diagnosis); 15,4/2 (probable).

c Milk allergy prevalence excluding patients reporting exclusively abdominal symptoms (%/n): 10.9/13 (general); 8.8/5 (medical diagnosis); 11.4/4 (probable)

Almost half (47.9%) of the included participants with self-reported FA reported that their allergic symptoms were associated with more than 1 food item. This proportion was higher in the self-reported FA medical diagnosis and probable FA groups (Table 3; Suppl Table 1). No particular combination of 2 or more foods was observed that could help distinguish between cross-reactions, culturally related food groups or true allergies to distinct foods in this group of patients with auto-reported multiple food allergies.

### Foods involved and clinical manifestations

Approximately two-thirds (62%) of elderly Brazilians reporting FA associated their reactions with at least 1 of the 8 major food groups responsible for most food allergies (milk, eggs, fish, shellfish, tree nuts, peanuts, wheat and soybeans).

The foods most frequently associated with FA symptoms in the studied population were shellfish, fresh fruits (including latex group fruits such as kiwi, banana, mango, papaya, figs, and tomatoes, as well as others not in the latex group), and milk (Fig. 1, Suppl Table 2). The relative frequency of each food varied depending on the criterion used in the definition of FA (Table 3, Suppl Table 2): while shellfish, fresh fruits and milk were the most commonly reported food items in the 3 groups, shellfish was reported by less than half participants with self-reported FA (46.2%) *vis-à-vis* more than two-thirds (68.6%) of those with probable FA.

**Fig. 1:**
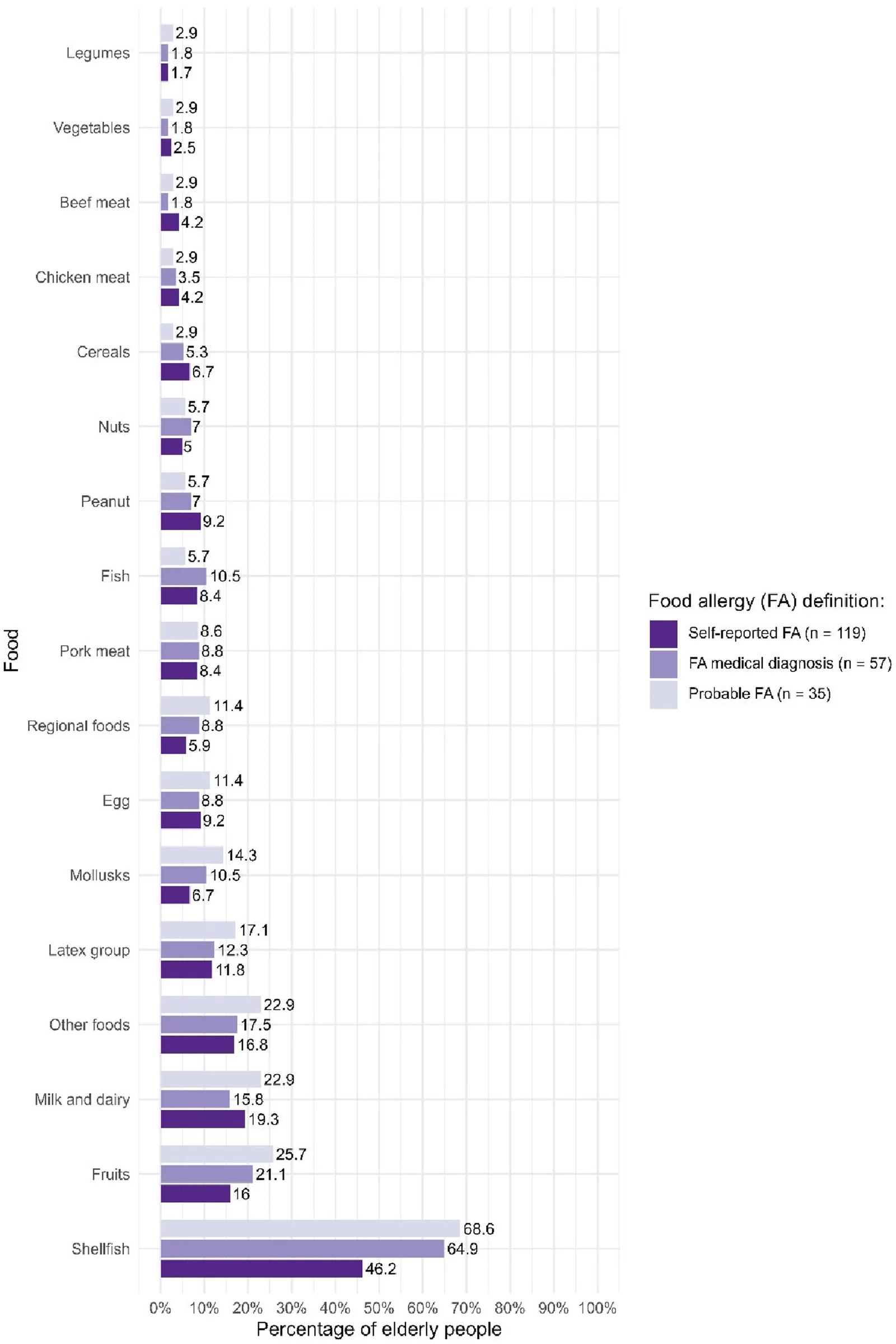
Foods most frequently associated with symptoms of food allergy

Considering all those with self-reported FA, the most reported clinical manifestations were urticaria, gastrointestinal (GI) symptoms and oral allergy symptoms (Table 3, Suppl Table 3). In the self-reported FA medical diagnosis and probable FA groups (Table 3, Suppl Tables 4 and 5), the most frequently reported symptoms were urticaria, respiratory symptoms and GI symptoms. The consumption of shellfish was the most frequently mentioned food in these 2 groups.

If we exclude patients reporting exclusively abdominal symptoms associated with the ingestion of milk and dairy products, the prevalence of self-reported milk allergy decreases by half, regardless of the used criteria (Table 3). Considering patients with self-reported FA to fresh fruits, only 15%–18% (depending on the criteria used) reported isolated OAS symptoms (Table 3).

The prevalence of self-reported food-induced anaphylaxis (FIA) varied according to the criteria used in defining FA (5.5%–7.1%), and again, shellfish was the most frequently implicated food (Table 3). Considering the total population included in the study, the prevalence of FIA ranges from 0.39% to 1.0% in the elderly population evaluated.

In the univariable and multivariable Poisson log-linear regression models, we found a significant association between female sex, allergic comorbidities and a family history of allergy and the prevalence of FA in the studied population. However, no association was found in multivariate analysis between distinct geographical regions, age groups, or education levels (Tables 4 and 5).

**Table 4:** Association between sociodemographic and clinical characteristics and self-reported Food Allergy (FA) in the elderly in Brazil: univariate analysis.

	General prevalence			FA medical diagnosis			Probable FA		
	(n = 119)			(n = 57)			(n = 35)		
	PR	95%CI	p-value	PR	95%CI	p-value	PR	95%CI	p-value
**Distribution across brazilian regions**			0.11			0.23			0.78
North	2.83	(1.24–6.44)	0.01	3.68	(1.14–11.84)	0.02	3.00	(0.48–18.67)	0.23
Northeast	1.31	(0.85–2.02)	0.20	0.77	(0.34–1.75)	0.53	0.83	(0.30–2.33)	0.73
Central-west	0.99	(0.49–1.99)	0.98	1.10	(0.41–2.92)	0.83	0.90	(0.22–3.65)	0.88
Southeast	1	–	–	1	–	–	1	–	–
South	1.30	0.47–3.59)	0.60	0.85	(0.12–5.71)	0.86	1.38	(0.20–9.44)	0.73
**Female sex**	1.62	(1.08–2.44)	0.01	2.31	(1.15–4.61)	0.01	2.09	(0.88–4.95)	0.09
**Age group**			0.15			0.24			0.60
60-69	1.27	(0.75–2.13)	0.36	1.48	(0.64–3.39)	0.35	1.72	(0.52–5.65)	0.36
70-79	0.88	(0.50–1.57)	0.68	0.94	(0.37–2.34)	0.89	1.37	(0.39–4.81)	0.61
≥80	1	–	–	1	–	–	1	–	–
**Level of education**			0.71			0.10			0.20
No education/elementary school	1	–	–	1	–	–	1	–	–
Middle/High school	1.09	(0.74–1.61)	0.63	1.83	(0.94–3.54)	0.07	1.93	(0.81–4.61)	0.13
Higher education	1.19	(0.78–1.81)	0.41	2.05	(1.02–4.10)	0.04	2.22	(0.89–5.52)	0.08
Allergic comorbidities	2.02	(1.33–3.07)	0.001	2.40	(1.24–4.66)	0.009	2.47	(1.04–5.86)	0.04
Family history of allergy	2.12	(1.48–3.05)	<0.001	2.72	(1.52–4.87)	0.001	2.56	(1.22–5.37)	0.01

PR: prevalence ratio; CI: confidence interval; p: p-value

**Table 5:** Association between sociodemographic and clinical characteristics and self-reported Food Allergy (FA) in the elderly in Brazil: multivariate analysis.

	General prevalence (n = 119)			FA medical diagnosis (n = 57)			Probable FA (n = 35)		
	PR	95%CI	p-value	PR	95%CI	p-value	PR	95%CI	p-value
Female sex	1.51	(1.01–2.25)	0.04	2.22	(1.12–4.41)	0.02			
Allergic comorbidities	1.62	(1.07–2.46)	0.02						
Family history of allergy	1.86	(1.30–2.67)	0.001	2.65	(1.48–4.75)	0.001	2.56	(1.22–5.37)	0.01

PR: prevalence ratio; CI: confidence interval. Allergic comorbidities: at least 1 of the 4 comorbidities (asthma, rhinitis, conjunctivitis and skin allergy), versus absence of all 4 comorbidities

## Discussion

In this non-probability, clinic-based sample of elderly adults (≥60 years) seeking allergy care in Brazil, the lifetime prevalence of self-reported FA is high, ranging from 5.5% to 18.8%, depending on the criteria used. Similar to other studies in adults, our data show that only about half of the Brazilian elderly with self-reported FA had a previous physician-confirmed diagnosis (9%).^14^

The use of distinct criteria (from most comprehensive to most restrictive) is an attempt to minimise overestimation of the true prevalence of FA in our study population (elderly individuals seeking allergy care), as our data rely on self-reports. As in other epidemiological studies, our definition of probable FA is based on the combination of responses to the self-completed questionnaire, rather than on a food challenge.^23^^,^^24^ Otherwise, in a country as large as Brazil (where there are significant regional disparities in access to health resources), it would be challenging to generate population-based prevalence estimates of FA in the elderly.

Few studies have specifically evaluated and/or reported the prevalence of food allergy among the elderly. Gupta et al,^14^ evaluating the prevalence of self-reported FA in North American adults, found a prevalence of “current FA” of 8.8% among patients aged 60 years or older. Lozoya-Ibanéz et al,^12^ in a cross-sectional study in Portugal, observed that the prevalence of self-reported FA in the elderly population evaluated was 4.4%. In comparison, Rentzos et al^15^ found a prevalence of self-reported FA of 19.1% in a cross-sectional study in Sweden.

Similar to other studies about self-reported FA in adults and the elderly, shellfish was the food most frequently reported as causing FA in Brazilian elderly, independently of the adopted FA definition.^8^^,^^25^ On the other hand, adverse reactions to milk and dairy products in adulthood are more likely to be associated with lactose intolerance than with FA. In our study, milk was the third food most associated with self-reported FA, predominantly presenting with GI symptoms, reinforcing the possibility that we are dealing with cases of food intolerance. Disregarding patients with exclusively abdominal symptoms associated with the ingestion of cow's milk and dairy products (potentially excluding those with lactose intolerance and delayed reactions to milk), the prevalence of self-reported cow's milk allergy decreases by half in our studied population, reinforcing this possibility.

GI, cutaneous and oral allergy symptoms were the most frequently reported clinical manifestations in Brazilian elderly, similar to those reported in other studies.^26^ However, regarding oral allergy symptoms, and unlike what is observed in Europe and the USA, pollen sensitisation is either not observed in most parts of Brazil (except for some states in the South Region),^27^ or is underdiagnosed,^28^ making the observation of oral symptoms as being associated with a cross-reaction between pollen sensitisation and fresh fruit intake intriguing in our population (only 15%–18% of patients with fresh fruit allergy included in the study reported isolated OAS symptoms). This finding needs to be confirmed in future studies involving the determination of specific IgE levels and oral challenge tests, as some elderly individuals may interpret the sensation of “prickling” in the mouth upon ingestion of acidic fruits (notably pineapple) as being an “allergy”. Alternatively, sensitisation to tropical fruit tree pollens, which have not been adequately studied,^28^ could be responsible for symptoms of pollinosis and pollen-food allergy syndrome.

In contrast with the situation in childhood and young adults, food is considered the least common cause of anaphylaxis in the elderly (where drugs and Hymenoptera venom are more common elicitors).^29^ Previous studies have reported a prevalence of FIA in the elderly ranging from 1% to 11%, with the most common triggers being shellfish and seafood, mammalian meat, wheat and hazelnut.^29^, ^30^, ^31^ Data from the Brazilian Registry of Anaphylaxis indicate a predominance of reactions caused by drugs and insects in the elderly, with food accounting for 0.6% of the reported cases, predominantly involving wheat and seafood.^32^ In the evaluated elderly Brazilian population, the findings are consistent with those reported by others, with a prevalence of around 1%, and shellfish being the most common trigger for self-reported anaphylaxis. However, it is important to highlight that prevalence estimates may have been influenced by the patient's understanding of the term “anaphylaxis” and by potential interviewer bias.

Little is known about the relationship between sociodemographic and clinical characteristics and the prevalence of FA in the elderly. Our findings suggest that female sex, allergic comorbidities (mainly rhinitis) and family history of allergy are significantly associated with the prevalence of elderly FA. Similar to our findings, Hultquist et al^33^ showed that women had a higher risk of developing adult-onset FA and suggested a link between allergic rhinitis and adult-onset FA. However, we did not find any associations with distribution across different Brazilian regions, age groups or educational levels, differently from other studies.^24^^,^^26^

This study has limitations. Our data is self-reported, and no confirmatory tests for the diagnosis of FA were performed. Our questionnaire did not include a question about the age of onset of symptoms of FA (childhood, adulthood or elderly), making it impossible to phenotypically characterise FA in our population (ie, childhood- or adult/elderly-onset FA).^33^ In addition to lacking information on the age at symptom onset, the study cannot distinguish active (current) food allergy from a past history of food allergy with possible acquired tolerance.

The use of a convenience sampling method, a type of non-probabilistic sampling, was chosen due to limited human resources, which provided flexibility in determining the sample size. Our initial target was to include 865 elderly individuals, proportionally distributed across Brazil. However, we interviewed and included 632 patients (73%), distributed across 14 Brazilian states. Notably, most included patients were from the Southeast region. This region accounts for almost 50% of Brazil's elderly population, yet represents 77% of the sample included in our study. This is likely due to the higher concentration of allergists in the Southeast region, which means a potential selection bias in the study. However, the impact of this imbalance on the generalisability of our results is not expected to be very high, since (i) similar results were obtained in multilevel models with post-stratification correcting for this imbalance, and (ii) in the multivariable analysis, the geographical distribution of the population across different Brazilian regions was not significantly associated with the prevalence of FA.

As the pool of participants was recruited in the context of allergology consultations and the study participation acceptance rate was not formally calculated, the prevalence of FA may be overestimated, given that the participants are likely to have a higher predisposition to allergic diseases, heightened perception of allergic symptoms or prior allergology investigation. Importantly, multilevel modelling with post-stratification does not correct for this clinical selection bias. A questionnaire applied to the general population (or in primary health care patients) would likely yield lower prevalence rates than those observed in our study population. However, a study on the prevalence of FA in the elderly in a country as large as Brazil would not be feasible without the participation of allergists and their widespread presence throughout the national territory.

On the other hand, the study has several strengths. This is the first Brazilian/Latin American multicenter study to use a standardised methodology and encompass all regions of the country, assessing the lifetime prevalence of FA exclusively in the elderly (≥60 years old). Despite being based on self-reporting, the standardised interviewer-administered approach and the use of different definitions to characterise FA provided both a broader and a more specific view of the prevalence of FA among the elderly in Brazil.

## Conclusions

Regardless of the used criteria, in this non-probability, clinic-based sample of elderly individuals in Brazil, the lifetime prevalence of self-reported FA was high, ranging from 5.5% to 18.8%. In patients ≥60 years, FA appears to be associated with the female sex and personal or family history of allergy. Shellfish, fresh fruits and cow's milk were the most frequently reported food allergens.

## Authors' statement of contribution to the work

All listed authors have made substantial contributions to the manuscript, namely:

JLB, DS, FK, JRM, EM: conception and design of the work; acquisition of data; analysis and data interpretation; drafting the work; critical revision of the manuscript.

BSP, JLD, LTB, CLI: conception and design of the work; analysis and data interpretation; critical revision for important intellectual content.

All other authors: acquisition of data, critical revision of the manuscript.

All authors: final approval of the version to be published; agreement to be accountable for all aspects of the work in ensuring that questions related to the accuracy or integrity of any part of the work are appropriately investigated and resolved.

## Statement of confirmation of unpublished work

The authors confirm that this manuscript is original, has not been published before, and is not currently being considered for publication elsewhere.

## Disclosure statement about the use of generative artificial intelligence

The authors have nothing to disclose.

## Funding

The authors declare that no funding was received for the present study.

## Potential competing interests and financial disclosure

BSP, JRM, LTB, CLI, JLD and MGAP report no competing interests.

All other authors: members of Associação Brasileira de Alergia e Imunologia (ASBAI), a non-profit medical scientific association that covered the article processing charge (APC) for this manuscript.
