# Supplementary material for: Self-reported food allergy in the elderly – Lifetime prevalence and clinical features in older adults seeking allergy care in Brazil
Source: World Allergy Organ J. 2026 Sep 28;19(10):101472. doi: 10.1016/j.waojou.2026.101472 (PMC13651488; doi:10.1016/j.waojou.2026.101472)
Supplement: Supplementary file 1 [file mmc1.pdf]

Questionnaire on Food allergies in elderly Brazilians

Date of administration: ____/____/______

1) Sex

- Male
- Female

2) Highest educational level achieved by the elderly subject:

- No education
- Incomplete elementary education or equivalent
- Complete elementary education or equivalent
- Incomplete secondary education or equivalent
- Complete secondary education or equivalent
- Incomplete higher education or equivalent
- Complete higher education
- Postgraduate certificate
- Masters degree
- Doctorate

3) Age in years

[ ] Date of birth: ____/____/______

4) Would you like to answer the questionnaire?

- Yes
- No

5) Have you ever had an allergic reaction to any type of food?

- Yes
- No

If question 5 was answered "No", go directly to questions 15 and 16 and end the interview.

6) Which foods provoke or have provoked reactions? (multiple choice)

- Milk and dairy
- Eggs
- Fish
- Seafood other than mollusks (seafood or crustaceans: shrimp, crab, lobster, etc.)
- Mollusks (mussels, octopus, squid)
- Peanuts
- Dry fruit (cashews, Brazil nuts, almonds, pistachio, hazelnuts, walnuts, etc.)
- Latex group (kiwi, banana, mango, carica papaya, figs, tomatoes)
- Fruit (other than those in the latex group)
- Vegetables (sweet corn, potatoes carrots, collard, etc.). NB: corn is considered a vegetable when fresh and a cereal when the grains are dried
- Legumes (beans, chickpeas, peas, lentils, soy, etc.)
- Cereals (wheat, rye, barley, oats)
- Chicken
- Pork
- Beef
- Regional foods (manioc, yams, açaí, etc.)
- Others
- Don't remember

If "Others" was chosen, specify:

7) **What type of reaction did you have? (describe the symptoms provoked against the foods that caused them. If subject doesn't remember, write "Don't remember")**
NB: more than one symptom can be endorsed per food.
OAS (Oral Allergy Syndrome)

	Urticaria/ Angioedema	Contact Dermatitis	OAS	Ocular	Nasal
Milk and dairy					
Eggs					
Fish					
Seafood					
Mollusks					
Peanuts					
Dry fruit					
Latex group					
Fruit (other than those in the latex group)					
Vegetables					
Legumes					
Cereals					
Chicken					
Pork					
Beef					
Regional foods					
Others					
Don't remember/ Don't know					

	Respiratory	Abdominal	Anaphylactic shock	Others
Milk and dairy				
Eggs				
Fish				
Seafood				
Mollusks				
Peanuts				
Dry fruit				
Latex group				
Fruit (other than those in the latex group)				
Vegetables				
Legumes				
Cereals				
Chicken				
Pork				
Beef				
Regional foods				
Others				
Don't remember/ Don't know				

8) **How long did it take for reactions to occur after ingestion of the food? (If necessary, specify per food)**
- Less than 30 min
- 30 min to less than 2 hours
- 2 hours to 24 hours
- More than 24 hours
- Don't remember

9) **Did you need medical treatment? (If necessary, specify per food)**
- Yes
- No

10) **If question 9 was answered "Yes", where were you treated?**
- Hospital emergency department
- SAMU / UPA
- Health center during first 24 hours
- Family physician after 24 hours
- Virtual or on-line consultation (telemedicine)
- Seen by a specialist
- Self-medication. Specify medications used: ____________________
- I don't remember where I was treated

11) **How many similar episodes have you had with the same food? (If necessary, specify per food)**
- Only 1 episode
- 2 to 5 episodes
- More than 5 episodes
- Don't remember

12) **How long since you last had a reaction? (If necessary, specify per food)**
- Less than 1 month
- 1 to 6 months
- More than 6 months to 1 year
- 1 year to 5 years
- More than 5 years
- Don't remember

13) **Have you ever been diagnosed with food allergy by a physician?**
- Yes
- No

14) **Have you ever had a consultation with an allergy specialist?**
- Yes
- No

15) **Other than reactions to food, do you have any type of allergic disease? (multiple choice)**
- Asthma (coughing, wheezing, shortness of breath)
- Rhinitis (sneezing, runny nose, itching nose, blocked nose)
- Conjunctivitis (eyes watering, itching, reddened)
- Allergy cutaneous (eczema, itching, scaling, urticaria)
- No
- Others (specify): ____________________

16) **Does anyone in your family have an allergic disease? (multiple choice)**
- Mother or father
- Brother or sister
- Grandparents
- Biological uncles
- Cousins
- Children
- No
- Others (specify): ____________________

Thank you very much for answering this questionnaire!

Supplementary Figure 1. Questionnaire on food allergies in elderly Brazilians

Boechat JL, Moraes JR, Taborda-Barata L, Lozoya-Ibáñez C, Sarinho ESC, Solé D. Self-reported food allergy among older Brazilians: prevalence and clinical characteristics – a study protocol. Arq Asma Alerg Imunol. 2022;6(4):483-90. http://dx.doi.org/10.5935/2526-5393.20220055
