# Supplementary material for: Self-reported food allergy in the elderly – Lifetime prevalence and clinical features in older adults seeking allergy care in Brazil
Source: World Allergy Organ J. 2026 Sep 28;19(10):101472. doi: 10.1016/j.waojou.2026.101472 (PMC13651488; doi:10.1016/j.waojou.2026.101472)
Supplement: Supplementary file 2 [file mmc2.pdf]

Supplemental Table 1: Patients according number of foods associated with food allergy (FA) by gender

Number of foods implicated	Self-reported FA				FA medical diagnosis				Probable FA			
	Male (n=25)	Female (n=94)	Total (n=119)	p-value*	Male (n=9)	Female (n=48)	Total (n=57)	p-value*	Male (n=6)	Female (n=29)	Total (n=35)	p-value
One	18 (72.0)	42 (44.7)	60 (50.4)		6 (66.7)	17 (35.4)	23 (40.4)		4 (66.7)	7 (24.1)	11 (31.4)	
Two or more	7 (28.0)	50 (53.2)	57 (47.9)	0.02	3 (33.3)	30 (62,5)	33 (57.9)	0.13	2 (33.3)	22 (75.9)	24 (68.6)	0.06
Not informed	0	2 (2.1)	2 (1.7)		0	1 (2.1)	1 (1.8)		-	-	-	

*In Fisher's Exact test, non-informants were not included

Supplemental Table 2: Distribution according to food allergy (FA) definition, type of food involved, and gender

Type of food implicated in the reported reaction	Self-reported FA, n(%)				FA medical diagnosis, n(%)				Probable FA, n(%)			
	Male (n=25)	Female (n=94)	Total (n=119)	p-value	Male (n=9)	Female (n=48)	Total (n=57)	p-value	Male (n=6)	Female (n=29)	Total (n=35)	p-value
Milk/dairy products	2 (8.0)	21 (22.3)	23 (19.3)	0.15	1 (11.1)	8 (16.7)	9 (15.8)	1.00	1 (16.7)	7 (24.1)	8 (22.9)	1.00
Egg	1 (4.0)	10 (10.6)	11 (9.2)	0.45	0	5 (10.4)	5 (8.8)	0.58	0	4 (13,8)	4 (11.4)	1.00
Fish	1 (4.0)	9 (9.6)	10 (8.4)	0.68	0	6 (12.5)	6 (10.5)	0.57	0	2 (6.9)	2 (5.7)	1.00
Shellfish	12 (48.0)	43 (45.7)	55 (46.2)	1.00	7 (77.8)	30 (62.5)	37 (64.9)	0.47	4 (66.7)	20 (69.0)	24 (68.6)	1.00
Mollusks	3 (12.0)	5 (5.3)	8 (6.7)	0.36	2 (22.5)	4 (8.3)	6 (10.5)	0.23	1 (16.7)	4 (13.8)	5 (14.3)	1.00
Peanuts	1 (4.0)	10 (10.6)	11 (9.2)	0.45	0	4 (8.3)	4 (7.0)	1.00	0	2 (6.9)	2 (5.7)	1.00
Nuts	1 (4.0)	5 (5.3)	6 (5.0)	1.00	1 (11.1)	3 (6.2)	4 (7.0)	0.50	1 (16.7)	1 (3.4)	2 (5.7)	0.31
Latex group	3 (12.0)	11 (11.7)	14 (11.8)	1.00	1 (11.1)	6 (12.5)	7 (12.3)	1.00	1 (16.7)	5 (17.2)	6 (17.1)	1.00
Fruits	2 (8.0)	17 (18.1)	19 (16.0)	0.35	0	12 (25.0)	12 (21.1)	0.18	0	9 (31.0)	9 (25.7)	0.30
Vegetables	1 (4.0)	2 (2.1)	3 (2.5)	0.51	0	1 (2.1)	1 (1.8)	1.00	0	1 (3.4)	1 (2.9)	1.00
Legumes	0	2 (2.1)	2 (1.7)	1.00	0	1 (2.1)	1 (1.8)	1.00	0	1 (3.4)	1 (2.9)	1.00
Cereals	1 (4.0)	7 (7.4)	8 (6.7)	1.00	0	3 (6.2)	3 (5.3)	1.00	0	1 (3.4)	1 (2.9)	1.00
Chicken meat	0	5 (5.3)	5 (4.2)	0.58	0	2 (4.2)	2 (3.5)	1.00	0	1 (3.4)	1 (2.9)	1.00
Pork meat	1 (4.0)	9 (9.6)	10 (8.4)	0.68	0	5 (10.4)	5 (8.8)	0.58	0	3 (10.3)	3 (8.6)	1.00
Beef	1 (4.0)	4 (4.3)	5 (4.2)	1.00	0	1 (2.1)	1 (1.8)	1.00	0	1 (3.4)	1 (2.9)	1.00
Regional foods	0	7 (7.4)	7 (5.9)	0.34	0	5 (10.4)	5 (8.8)	0.58	0	4 (13.8)	4 (11.4)	1.00
Other foods	5 (20.0)	15 (16.0)	20 (16.8)	0.76	1 (11.1)	9 (18.8)	10 (17.5)	1.00	1 (16.7)	7 (24.1)	8 (22.9)	1.00

p-value of Fisher's Exact test

"Other foods" represents a heterogeneous group of foods mentioned individually: saffron, rosemary, colorants and stronger spices, red dye, cocoa, cinnamon, lemongrass, fennel, tea, coriander, condiments, ginger, mint, tomato sauce, mortadella, red gelatin, ham, pepper, propolis, sausage, frankfurter.

Supplemental Table 3. Self-reported symptoms associated with each food type and gender (general prevalence)

Type of food implicated in the reported reaction	Gender	Self-reported symptoms (n=119), n (%)*								
		Urticaria /Angioedema	Contact dermatitis	OAS	Ocular	Nasal	Respiratory	Abdominal	Anaphylaxis	Other
Milk and dairy products	Total (n=23)	6 (26.1)	0	0	2 (8.7)	2 (8.7)	1 (4.3)	16 (69.6)	1 (4.3)	6 (26.1)
	Female (n=21)	6 (28.6)	0	0	2 (9.5)	2 (9.5)	1 (4.8)	15 (71.4)	1 (4.8)	5 (23.8)
	Male (n=2)	0	0	0	0	0	0	1 (50.0)	0	1 (50.0)
Egg	Total (n=11)	6 (54.5)	0	0	0	1 (9.1)	1 (9.1)	6 (54.5)	1 (9.1)	1 (9.1)
	Female (n=10)	5 (50.0)	0	0	0	1 (10.0)	1 (10.0)	6 (60.0)	1 (10.0)	1 (10.0)
	Male (n=1)	1 (100)	0	0	0	0	0	0	0	0
Fish	Total (n=10)	7 (70.0)	0	3 (30.0)	2 (20.0)	3 (30.0)	2 (20.0)	2 (20.0)	1 (10.0)	1 (10.0)
	Female (n=9)	7 (77.8)	0	2 (22.2)	2 (22.2)	3 (33.3)	2 (22.2)	2 (22.2)	1 (11.1)	1 (11.1)
	Male (n=1)	0	0	1 (100)	0	0	0	0	0	0
Shellfish	Total (n=55)	49 (89.1)	3 (5.5)	8 (14.5)	7 (12.7)	4 (7.3)	8 (14.5)	9 (16.4)	5 (9.1)	1 (1.8)
	Female (n=43)	38 (88.4)	2 (4.7)	6 (14.0)	6 (14.0)	2 (4.7)	7 (16.3)	5 (11.6)	3 (7.0)	1 (2.3)
	Male (n=12)	11 (91.7)	1 (8.3)	2 (16.7)	1 (8.3)	2 (16.7)	1 (8.3)	4 (33.3)	2 (16.7)	0
Mollusks	Total (n=8)	7 (87.5)	1 (12.5)	0	1 (12.5)	0	0	0	0	1 (12.5)
	Female (n=5)	5 (100)	0	0	0	0	0	0	0	0
	Male (n=3)	2 (66.7)	1 (33.3)	0	1 (33.3)	0	0	0	0	1 (33.3)
Peanuts	Total (n=11)	7 (63.6)	1 (9.1)	0	5 (45.5)	1 (9.1)	1 (9.1)	3 (27.3)	1 (9.1)	2 (18.2)
	Female (n=10)	7 (70.0)	1 (10.0)	0	5 (50.0)	1 (10.0)	1 (10.0)	2 (20.0)	1 (10.0)	1 (10.0)
	Male (n=1)	0	0	0	0	0	0	1 (100)	0	1 (100)
Nuts	Total (n=6)	4 (66.7)	0	1 (16.7)	1 (16.7)	1 (16.7)	2 (33.3)	0	1 (16.7)	0
	Female (n=5)	4 (80.0)	0	1 (20.0)	1 (20.0)	1 (20.0)	1 (20.0)	0	1 (20.0)	0
	Male (n=1)	0	0	0	0	0	1 (100)	0	0	0
Latex group	Total (n=14)	7 (50.0)	1 (7.1)	5 (35.7)	2 (14.3)	2 (14.3)	2 (14.3)	3 (21.4)	2 (14.3)	1 (7.1)
	Female (n=11)	6 (54.5)	1 (9.1)	3 (27.3)	2 (18.2)	2 (18.2)	2 (18.2)	3 (27.3)	2 (18.2)	1 (9.1)
	Male (n=3)	1 (33.3)	0	2 (66.7)	0	0	0	0	0	0

Fruits	Total (n=19)	11 (57.9)	2 (10.5)	5 (26.3)	2 (10.5)	3 (15.8)	3 (15.8)	3 (15.8)	1 (5.3)	2 (10.5)
	Female (n=17)	9 (52.9)	2 (11.8)	5 (29.4)	2 (11.8)	3 (17.6)	3 (17.6)	3 (17.6)	1 (5.9)	2 (11.8)
	Male (n=2)	2 (100)	0	0	0	0	0	0	0	0
Vegetables	Total (n=3)	3 (100)	0	1 (33.3)	1 (33.3)	1 (33.3)	1 (33.3)	0	1 (33.3)	0
	Female (n=2)	2 (100)	0	1 (50.0)	1 (50.0)	1 (50.0)	1 (50.0)	0	1 (50.0)	0
	Male (n=1)	1 (100)	0	0	0	0	0	0	0	0
Legumes	Total (n=2)	0	0	0	0	0	0	2 (100)	0	0
	Female (n=2)	0	0	0	0	0	0	2 (100)	0	0
	Male (n=0)	0	0	0	0	0	0	0	0	0
Cereals	Total (n=8)	4 (50.0)	0	2 (25.0)	0	1 (12.5)	1 (12.5)	4 (50.0)	1 (12.5)	0
	Female (n=7)	3 (42.9)	0	1 (14.3)	0	0	1 (14.3)	4 (57.1)	1 (14.3)	0
	Male (n=1)	1 (100)	0	1 (100)	0	1 (100)	0	0	0	0
Chicken meat	Total (n=5)	3 (60.0)	0	1 (20.0)	0	1 (20.0)	1 (20.0)	1 (20.0)	0	0
	Female (n=5)	3 (60.0)	0	1 (20.0)	0	1 (20.0)	1 (20.0)	1 (20.0)	0	0
	Male (n=0)	0	0	0	0	0	0	0	0	0
Pork meat	Total (n=10)	7 (70.0)	0	2 (20.0)	2 (20.0)	2 (20.0)	1 (10.0)	2 (20.0)	0	1 (10.0)
	Female (n=9)	7 (77.8)	0	1 (11.1)	2 (22.2)	2 (22.2)	1 (11.1)	2 (22.2)	0	1 (11.1)
	Male (n=1)	0	0	1 (100)	0	0	0	0	0	0
Beef	Total (n=5)	2 (40.0)	0	0	0	1 (20.0)	0	3 (60.0)	0	2 (40.0)
	Female (n=4)	2 (50.0)	0	0	0	1 (25.0)	0	3 (75.0)	0	1 (25.0)
	Male (n=1)	0	0	0	0	0	0	0	0	1 (100)
Regional foods	Total (n=7)	2 (28.6)	2 (28.6)	1 (14.3)	0	0	2 (28.6)	2 (28.6)	2 (28.6)	1 (14.3)
	Female (n=7)	2 (28.6)	2 (28.6)	1 (14.3)	0	0	2 (28.6)	2 (28.6)	2 (28.6)	1 (14.3)
	Male (n=0)	0	0	0	0	0	0	0	0	0
Other foods	Total (n=20)	15 (75.0)	0	5 (25.0)	5 (25.0)	3 (15.0)	2 (10.0)	2 (10.0)	3 (15.0)	0
	Female (n=15)	11 (73.3)	0	4 (26.7)	5 (33.3)	3 (20.0)	2 (13.3)	2 (13.3)	3 (20.0)	0
	Male (n=5)	4 (80.0)	0	1 (20.0)	0	0	0	0	0	0

*As each participant may report different symptoms associated with the same food, the final sum may be different from 100%

OAS: oral allergic syndrome

Supplemental Table 4: Self-reported symptoms associated with each food type and gender (food allergy medical diagnosis)

Type of food implicated in the reported reaction	Gender	Self-reported symptoms (n=57), n (%)*								
		Urticária /Angioedema	Contact dermatitis	OAS	Ocular	Nasal	Respiratory	Abdominal	Anaphylaxis	Other
Milk and dairy products	Total (n=9)	4 (44.4)	0	0	0	0	0	5 (55.6)	0	1 (11.1)
	Female (n=8)	4 (50.0)	0	0	0	0	0	4 (50.0)	0	1 (12.5)
	Male (n=1)	0	0	0	0	0	0	1 (50.0)	0	1 (50.0)
Egg	Total (n=5)	4 (80.0)	0	0	0	1 (20.0)	1 (20.0)	3 (60.0)	1 (20.0)	1 (20.0)
	Female (n=5)	4 (80.0)	0	0	0	1 (20.0)	1 (20.0)	3 (60.0)	1 (20.0)	1 (20.0)
	Male (n=0)	0	0	0	0	0	0	0	0	0
Fish	Total (n=6)	5 (83.3)	0	2 (33.3)	2 (33.3)	1 (16.7)	2 (33.3)	0	1 (16.7)	0
	Female (n=6)	5 (83.3)	0	2 (33.3)	2 (33.3)	1 (16.7)	2 (33.3)	0	1 (16.7)	0
	Male (n=0)	0	0	0	0	0	0	0	0	0
Shellfish	Total (n=37)	34 (91.9)	1 (2.7)	8 (21.6)	7 (18.9)	4 (10.8)	7 (18.9)	6 (16.2)	4 (10.8)	1 (2.7)
	Female (n=30)	27 (90.0)	1 (3.3)	6 (20.0)	6 (20.0)	2 (6.7)	6 (20.0)	5 (16.7)	3 (10.0)	1 (3.3)
	Male (n=7)	7 (100)	0	2 (28.6)	1 (14.3)	2 (28.3)	1 (14.3)	1 (14.3)	1 (14.3)	0
Mollusks	Total (n=6)	6 (100)	1 (16.7)	0	1 (16.7)	0	0	0	0	0
	Female (n=4)	4 (100)	0	0	0	0	0	0	0	0
	Male (n=2)	2 (100)	1 (50.0)	0	1 (50.0)	0	0	0	0	0
Peanuts	Total (n=4)	4 (100)	1 (25.0)	0	4 (100)	0	1 (25.0)	1 (25.0)	0	0
	Female (n=4)	4 (100)	1 (25.0)	0	4 (100)	0	1 (25.0)	1 (25.0)	0	0
	Male (n=0)	0	0	0	0	0	0	0	0	0
Nuts	Total (n=4)	3 (75.0)	0	0	0	0	2 (50.0)	0	1 (25.0)	0
	Female (n=3)	3 (100)	0	0	0	0	1 (33.3)	0	1 (33.3)	0
	Male (n=1)	0	0	0	0	0	1 (100)	0	0	0
Latex group	Total (n=7)	4 (57.1)	1 (14.3)	3 (42.9)	2 (28.6)	2 (28.6)	2 (28.6)	2 (28.6)	2 (28.6)	0
	Female (n=6)	4 (66.7)	1 (16.7)	2 (33.3)	2 (33.3)	2 (33.3)	2 (33.3)	2 (33.3)	2 (33.3)	0

	Male (n=1)	0	0	1 (100)	0	0	0	0	0	0
Fruits	Total (n=12)	7 (58.3)	1 (8.3)	4 (33.3)	1 (8.3)	2 (16.7)	3 (25.0)	2 (16.7)	1 (8.3)	1 (8.3)
	Female (n=12)	7 (58.3)	1 (8.3)	4 (33.3)	1 (8.3)	2 (16.7)	3 (25.0)	2 (16.7)	1 (8.3)	1 (8.3)
	Male (n=0)	0	0	0	0	0	0	0	0	0
Vegetables	Total (n=1)	1 (100)	0	0	0	0	0	0	0	0
	Feminino (n=1)	1 (100)	0	0	0	0	0	0	0	0
	Male (n=0)	0	0	0	0	0	0	0	0	0
Legumes	Total (n=1)	0	0	0	0	0	0	1 (100)	0	0
	Female (n=1)	0	0	0	0	0	0	1 (100)	0	0
	Male (n=0)	0	0	0	0	0	0	0	0	0
Cereals	Total (n=3)	2 (66.7)	0	1 (33.3)	0	0	1 (33.3)	1 (33.3)	1 (33.3)	0
	Feminino (n=3)	2 (66.7)	0	1 (33.3)	0	0	1 (33.3)	1 (33.3)	1 (33.3)	0
	Male (n=0)	0	0	0	0	0	0	0	0	0
Chicken meat	Total (n=2)	0	0	1 (50.0)	0	0	1 (50.0)	0	0	0
	Female (n=2)	0	0	1 (50.0)	0	0	1 (50.0)	0	0	0
	Male (n=0)	0	0	0	0	0	0	0	0	0
Pork meat	Total (n=5)	5 (100)	0	0	2 (40.0)	1 (20.0)	1 (20.0)	0	0	0
	Female (n=5)	5 (100)	0	0	2 (40.0)	1 (20.0)	1 (20.0)	0	0	0
	Male (n=0)	0	0	0	0	0	0	0	0	0
Beef	Total (n=1)	0	0	0	0	0	0	1 (100)	0	0
	Feminino (n=1)	0	0	0	0	0	0	1 (100)	0	0
	Male (n=0)	0	0	0	0	0	0	0	0	0
Regional foods	Total (n=5)	1 (20.0)	2 (40.0)	1 (20.0)	0	0	2 (40.0)	1 (20.0)	2 (40.0)	1 (20.0)
	Female (n=5)	1 (20.0)	2 (40.0)	1 (20.0)	0	0	2 (40.0)	1 (20.0)	2 (40.0)	1 (20.0)
	Male (n=0)	0	0	0	0	0	0	0	0	0
Other foods	Total (n=10)	9 (90.0)	0	3 (30.0)	5 (50.0)	3 (30.0)	2 (20.0)	1 (10.0)	2 (20.0)	0
	Female (n=9)	8 (88.9)	0	3 (33.3)	5 (55.6)	3 (33.3)	2 (22.2)	1 (11.1)	2 (22.2)	0
	Male (n=1)	1 (100)	0	0	0	0	0	0	0	0

*As each participant may report different symptoms associated with the same food, the final sum may be different from 100%. OAS: oral allergic syndrome

Supplemental Table 5: Self-reported symptoms associated with each food type and gender (Probable food allergy)

Type of food implicated in the reported reaction	Gender	Self-reported symptoms (n=35), n (%)*								
		Urticária /Angioedema	Contact dermatitis	OAS	Ocular	Nasal	Respiratory	Abdominal	Anaphylaxis	Other
Milk and dairy products	Total (n=8)	3 (37.5)	0	0	0	0	0	5 (62.5)	0	1 (12.5)
	Female (n=7)	3 (42.9)	0	0	0	0	0	4 (57.1)	0	1 (14.3)
	Male (n=1)	0	0	0	0	0	0	1 (100)	0	0
Egg	Total (n=4)	3 (75.0)	0	0	0	1 (25.0)	1 (25.0)	2 (50.0)	1 (25.0)	1 (25.0)
	Female (n=4)	3 (75.0)	0	0	0	1 (25.0)	1 (25.0)	2 (50.0)	1 (25.0)	1 (25.0)
	Male (n=0)	0	0	0	0	0	0	0	0	0
Fish	Total (n=2)	2 (100)	0	1 (50.0)	1 (50.0)	1 (50.0)	0	0	0	0
	Female (n=2)	2 (100)	0	1 (50.0)	1 (50.0)	1 (50.0)	0	0	0	0
	Male (n=0)	0	0	0	0	0	0	0	0	0
Shellfish	Total (n=24)	21 (87.5)	1 (4.2)	7 (29.2)	3 (12.5)	4 (16.7)	6 (25.0)	6 (25.0)	3 (12.5)	1 (4.2)
	Female (n=20)	17 (85.0)	1 (5.0)	5 (25.0)	3 (15.0)	2 (10.0)	5 (25.0)	5 (25.0)	2 (10.0)	1 (5.0)
	Male (n=4)	4 (100)	0	2 (50.0)	0	2 (50.0)	1 (25.0)	1 (25.0)	1 (25.0)	0
Mollusks	Total (n=5)	5 (100)	1 (20.0)	0	0	0	0	0	0	0
	Female (n=4)	4 (100)	0	0	0	0	0	0	0	0
	Male (n=1)	1 (100)	1 (100)	0	0	0	0	0	0	0
Peanuts	Total (n=2)	2 (100)	1 (50.0)	0	2 (100)	0	1 (50.0)	1 (50.0)	0	0
	Female (n=2)	2 (100)	1 (50.0)	0	2 (100)	0	1 (50.0)	1 (50.0)	0	0
	Male (n=0)	0	0	0	0	0	0	0	0	0
Nuts	Total (n=2)	1 (50.0)	0	0	0	0	2 (100)	0	1 (50.0)	0
	Female (n=1)	1 (100)	0	0	0	0	1 (100)	0	1 (100)	0
	Male (n=1)	0	0	0	0	0	1 (100)	0	0	0
Latex group	Total (n=6)	3 (50.0)	1 (16.7)	3 (50.0)	2 (33.3)	2 (33.3)	2 (33.3)	2 (33.3)	2 (33.3)	0
	Female (n=5)	3 (60.0)	1 (20.0)	2 (40.0)	2 (40.0)	2 (40.0)	2 (40.0)	2 (40.0)	2 (40.0)	0

	Male (n=1)	0	0	1 (100)	0	0	0	0	0	0
Fruits	Total (n=9)	6 (66.7)	0	2 (22.2)	1 (11.1)	2 (22.2)	3 (33.3)	2 (22.2)	1 (11.1)	1 (11.1)
	Female (n=9)	6 (66.7)	0	2 (22.2)	1 (11.1)	2 (22.2)	3 (33.3)	2 (22.2)	1 (11.1)	1 (11.1)
	Male (n=0)	0	0	0	0	0	0	0	0	0
Vegetables	Total (n=1)	1 (100)	0	0	0	0	0	0	0	0
	Female (n=1)	1 (100)	0	0	0	0	0	0	0	0
	Male (n=0)	0	0	0	0	0	0	0	0	0
Legumes	Total (n=1)	0	0	0	0	0	0	1 (100)	0	0
	Female (n=1)	0	0	0	0	0	0	1 (100)	0	0
	Male (n=0)	0	0	0	0	0	0	0	0	0
Cereals	Total (n=1)	0	0	0	0	0	0	1 (100)	0	0
	Female (n=1)	0	0	0	0	0	0	1 (100)	0	0
	Male (n=0)	0	0	0	0	0	0	0	0	0
Chicken meat	Total (n=1)	0	0	1 (100)	0	0	0	0	0	0
	Female (n=1)	0	0	1 (100)	0	0	0	0	0	0
	Male (n=0)	0	0	0	0	0	0	0	0	0
Pork meat	Total (n=3)	3 (100)	0	0	1 (33.3)	1 (33.3)	1 (33.3)	0	0	0
	Female (n=3)	3 (100)	0	0	1 (33.3)	1 (33.3)	1 (33.3)	0	0	0
	Male (n=0)	0	0	0	0	0	0	0	0	0
Beef	Total (n=1)	0	0	0	0	0	0	1 (100)	0	0
	Female (n=1)	0	0	0	0	0	0	1 (100)	0	0
	Male (n=0)	0	0	0	0	0	0	0	0	0
Regional foods	Total (n=4)	1 (25.0)	1 (25.0)	1 (25.0)	0	0	2 (50.0)	1 (25.0)	2 (50.0)	1 (25.0)
	Female (n=4)	1 (25.0)	1 (25.0)	1 (25.0)	0	0	2 (50.0)	1 (25.0)	2 (50.0)	1 (25.0)
	Male (n=0)	0	0	0	0	0	0	0	0	0
Other foods	Total (n=8)	7 (87.5)	0	3 (37.5)	3 (37.5)	3 (37.5)	2 (25.0)	1 (12.5)	2 (25.0)	0
	Female (n=7)	6 (85.7)	0	3 (42.9)	3 (42.9)	3 (42.9)	2 (28.6)	1 (14.3)	2 (28.6)	0
	Male (n=1)	1 (100)	0	0	0	0	0	0	0	0

*As each participant may report different symptoms associated with the same food, the final sum may be different from 100%. OAS: oral allergic syndrome
